# Bridging the Gap: Guideline Adherence in IgG4‐Related Digestive Disease

**DOI:** 10.1002/ueg2.70052

**Published:** 2025-05-22

**Authors:** Katja Kilani, Alexander Kleger

**Affiliations:** ^1^ Division of Interdisciplinary Pancreatology Department of Internal Medicine I Ulm University Hospital Ulm Germany; ^2^ Institute for Molecular Oncology and Stem Cell Biology Ulm University Hospital Ulm Germany

## Abstract

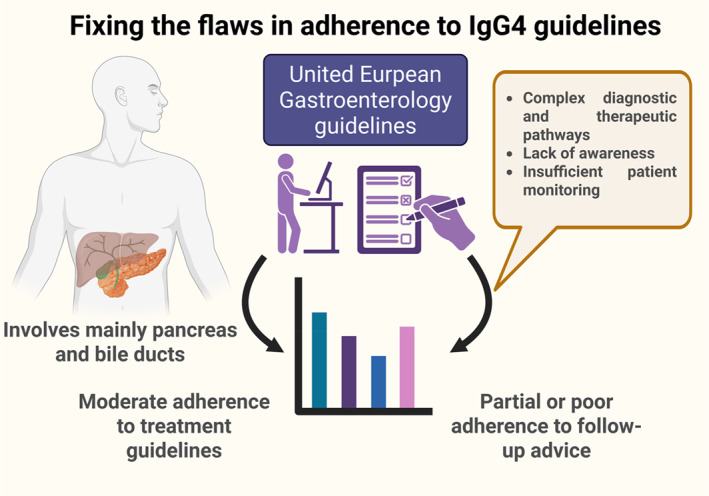

## 1 Introduction

Immunoglobulin G4‐related disease (IgG4‐RD) is a rare, immune‐mediated fibroinflammatory condition characterized by tumor‐like lesions, a lymphoplasmacytic infiltrate rich in IgG4‐positive plasma cells, and often elevated serum IgG4 levels. While it can affect virtually any organ system, the pancreas and bile ducts are among the most frequently involved sites in gastrointestinal practice [1, 2]. First‐line treatment relies on glucocorticoids, which are effective but prone to relapse and long‐term toxicity. B cell depletion—especially with anti‐CD20 or CD19 antibodies—has emerged as a promising alternative, and with inebilizumab from the MITIGATE trial, the first FDA‐approved therapy is entering clinical use [3]. Against the backdrop of diagnostic complexity and evolving therapeutic options, clear and widely implemented clinical guidelines are essential to ensure high‐quality, standardized care for patients with IgG4‐RD.

Guidelines represent more than expert consensus—they are a blueprint for delivering evidence‐based, equitable, and timely care. This principle is especially critical in rare, complex conditions like immunoglobulin G4‐related disease (IgG4‐RD), where diagnosis is challenging and missteps can result in irreversible organ damage or unnecessary interventions. In this issue of *United European Gastroenterology Journal*, Vujasinovic et al. report a pan‐European snapshot of clinical adherence to the 2020 UEG guidelines on the diagnosis and management of IgG4‐related digestive diseases [4, 5]. The findings offer both reassurance and a sobering reminder: even in specialized centers, implementation remains incomplete.

The study draws on data from 199 patients across 14 centers in 11 European countries, evaluated over a 3‐year period. While treatment protocols were followed in the majority of cases, particularly for autoimmune pancreatitis (AIP) and IgG4‐related cholangitis (IgG4‐C), gaps emerged in follow‐up and disease monitoring. Less than half of patients underwent follow‐up at the recommended 2–4 weeks, and only a minority of centers used the IgG4‐responder index to track disease activity.

These findings are striking given the profile of the participating centers—most were tertiary referral hospitals with a declared interest in IgG4‐RD. That even expert centers struggle with guideline implementation speaks to broader challenges in translating evidence into routine care. It also raises the question: if adherence is moderate here, what can be expected in less specialized environments?

From a methodological perspective, the study is notable for its real‐world scope and uniformity of data collection. It is, to our knowledge, the first multi‐national effort to benchmark clinical practice against the UEG IgG4‐RD guideline. Importantly, it highlights the uneven adoption not only of diagnostic criteria and therapeutic regimens but also of follow‐up and long‐term monitoring strategies—components that are essential for preventing relapses and avoiding unnecessary surgical procedures in this cancer‐mimicking condition.

## 2 Why Is Adherence Limited?

As the authors rightly note, the rarity and complexity of IgG4‐RD likely contribute to clinical uncertainty. Furthermore, the perceived burden of adhering to guideline‐recommended monitoring protocols, such as imaging at strict time intervals or structured responder indices, may deter busy clinicians in resource‐constrained settings. Systemic barriers such as limited access to advanced imaging, heterogeneity in health IT systems and the absence of multidisciplinary teams further compromise the implementation of guideline‐concordant care.

But there is also an opportunity embedded in these findings.

Firstly, the variability identified in this study could be uses as a foundation for the implementation of targeted educational interventions, especially in non‐specialist centers. Secondly, the authors propose the development of a centralized European database—a concept that could not only monitor adherence but also support continuous learning, feedback loops, and perhaps even real‐time clinical decision support.

These implications extend beyond IgG4‐RD. As European healthcare systems evolve toward personalized medicine, the success of such efforts will increasingly depend on the implementation of scientific principles.

## 3 How Do We Ensure That Excellent Guidelines Do Not Gather Dust?

This study reminds us that crafting recommendations is only the beginning; the hard work lies in embedding them into clinical practice. Importantly, the authors emphasize the potential of collaborative care models. Given the multisystemic nature of IgG4‐RD, gastroenterologists, hepatologists, radiologists, pathologists, rheumatologists, and immunologists must work in concert. Shared care pathways and referral networks can not only improve outcomes but may also serve as scalable models for other rare or complex disorders.

Finally, the editorial would be incomplete without addressing the patient perspective. IgG4‐RD often affects individuals at an older age, where comorbidities and frailty influence therapeutic decisions. Guideline adherence should be contextualized—not blindly applied. The aim should not be to achieve rigid uniformity, but rather to provide informed, patient‐centered care based on the best available evidence. In conclusion, the work by Vujasinovic et al. is a timely and important contribution. It affirms the value of the UEG guidelines, identifies critical gaps in their implementation, and offers a roadmap for improvement. Future efforts should prioritize education, system‐level support, and data‐driven feedback mechanisms. In a field where underdiagnosis, overtreatment, and misclassification remain common, closing the gap between guidance and practice is not just a quality issue—it's a clinical imperative.

## Conflicts of Interest

The authors declare no conflicts of interest.

## Data Availability

The data that support the findings of this study are available on request from the corresponding author. The data are not publicly available due to privacy or ethical restrictions.
